# A stable polymeric chain configuration producing high performance PEBAX-1657 membranes for CO_2_ separation†

**DOI:** 10.1039/c9na00170k

**Published:** 2019-05-14

**Authors:** Pankaj Sharma, Young-Jin Kim, Min-Zy Kim, Syed Fakhar Alam, Churl Hee Cho

**Affiliations:** Graduate School of Energy Science and Technology, Chungnam National University 99 Daehak-ro, Yuseong-gu Daejeon 34134 Republic of Korea sharmapankaj47@yahoo.com choch@cnu.ac.kr

## Abstract

Although PEBAX-1657 is one of the promising polymeric materials for selective CO_2_ separation, there remain many questions about the optimal polymeric structure and possibility of improving performance without adulterating its basic structure by impregnating inorganic fillers. In order to improve the gas separation performance, low thickness PEBAX membranes were synthesized under steady solvent evaporation conditions by keeping in mind that one of its segments (nylon 6) shows structural variance and molecular orientation with a change in the evaporation rate. Furthermore, phase pure zeolite nanocrystals with cubic (zeolite A) and octahedral (zeolite Y) shapes have been synthesized through liquid phase routes using microwave hydrothermal reactors. The average sizes of zeolite A and Y crystals are around 55 and 40 nm, respectively. The inspection of XRD, DSC and Raman shift of PEBAX membranes demonstrates the formation of a stable polymeric structure with an improved crystalline state which results in high CO_2_ permeability membranes. The CO_2_ permeability as well as diffusivity increase with a decrease in membrane thickness and reach a maximum value of 184.7 Barrer and 2.6 × 10^−6^ cm^2^ s^−1^, respectively. The as-fabricated pristine PEBAX membrane shows much better performance in terms of permeance (CO_2_ 184.7 Barrer), diffusivity (CO_2_ 2.6 × 10^−6^ cm^2^ s^−1^) and selectivity (CO_2_/N_2_ 59.7), which substantiate its promising prospects for CO_2_ capture. This exceptional performance of the pristine PEBAX membrane arises from the free volume generated during the steady polymerization. This reported approach for PEBAX membrane synthesis provides a direction in the design of membrane fabrication processes for economic CO_2_ separation.

## Abstract 2

By improving the polymeric structure and reforming the free-volume of pristine PEBAX films, the permeability, as well as CO_2_ selectivity, can be enhanced simultaneously.

## Graphical abstract

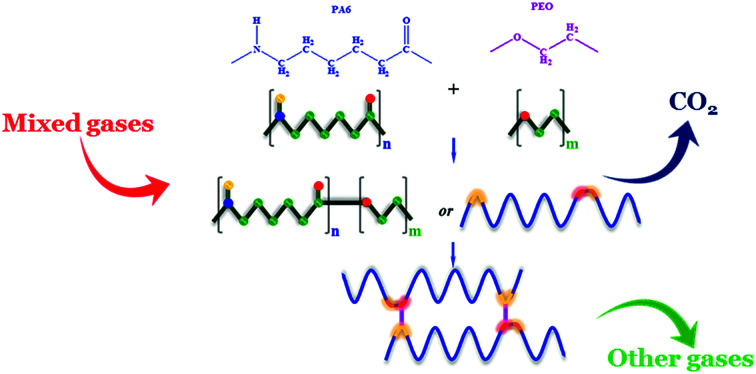

## 1. Introduction

Global warming is mainly caused by the emission of greenhouse gases and 72% of the total emitted greenhouse gases are carbon dioxide (CO_2_), 18% methane and 9% nitrous oxide (NO_*x*_). Therefore, CO_2_ emission is the primary cause of global warming, and human activities are mainly responsible for greenhouse gas emission into the earth environment.^1^ Thus, CO_2_ capture, separation, and storage have been considered as one of the most important technological efforts to combat the global warming challenge. Compared to well-known technologies for CO_2_ capture including amine scrubbing, solid adsorption, solvent adsorption, and cryogenic distillation, membrane separation is more promising due to its system compactness (small footprint), ease of operation, least environmental impact and exceptional reliability.^2–10^ Particularly, the amine scrubbing based post-combustion CO_2_ capture process has large human health and environmental concern because of solvent emissions during the processing. Furthermore, a high amount of energy is needed for CO_2_ desorption which results in efficiency loss in the case of amine-based scrubbing.

Inorganic and polymeric membranes are two major players for CO_2_ capture.^11^ Although, inorganic membranes have also been extensively investigated for gas separation due to their exceptional separation performance and incredible thermal and chemical stability, their cost and lack of processability are the major issues for their large-scale production. Furthermore, assembling of inorganic membranes into high packing density membrane modules is critical for the flue gas process. On the other hand, polymeric membranes have emerged as an ideal candidate for large scale processing plants.^12,13^ However, polymer-based membranes have a major drawback, the so-called permeability–selectivity trade-off, *i.e.*, either increase in permeability or decrease in selectivity or *vice versa*.^14^

To address these issues and to prepare novel membranes/materials, researchers made attempts to combine the merits of polymeric and inorganic materials to develop a new class of membranes.^14–23^ Poly(ether-*block*-amide) polymer commonly known as PEBAX is considered as one of the ideal polymeric materials for membrane-based separation of CO_2_ from flue gas and natural gas because of its comparatively high gas permeability as well as CO_2_ selectivity.^1,16,24–38^ PEBAX consists of two monomers polyethylene oxide (PEO) and polyamide (PA), where the earlier component provides flexibility and high CO_2_ permeability due to its high affinity with the polar CO_2_ molecule, while the latter component imparts mechanical strength to the membrane. As ether oxygen moieties have a significant affinity to acid gases, PEBAX is considered as one of the best candidates for CO_2_ separation from light gases.^24,32^ The perfect balance between permeability and CO_2_ selectivity can be achieved by increasing the free flow of gas molecules through the polymeric membrane without altering the surface structure of the polymer.

Polymer free-volume, the fraction of the volume not occupied by the electronic clouds of the polymer, plays an important role in the transport properties of low molecular weight species and gases. In other words, molecular transport through a dense polymer depends strongly on free volume; therefore, control over free-volume is thus important for the development of better membranes for a wide variety of applications such as gas separation, pharmaceutical purification, and energy storage.^13,15,24,39–43^ Free volume (*i.e.*, static voids created by inefficient chain packing or transient gaps generated by thermally induced chain segment rearrangements) presents diffusing molecules with a low resistance avenue for transport.^15,39,40,44–47^ Not only the overall amount of free-volume but also the distribution of the effective micropore size is likely to have a significant influence on polymer properties if the free-volume elements are interconnected. The use of inorganic fillers in mixed matrix membrane (MMM) is to influence the free volume.^15,24,40^ However, the addition of cost-ineffective nano-size inorganic fillers generally shows negative effects on the selectivity with little improvement in permeability. If the addition of nano-inorganic fillers is just to improve the permeability of the membrane, then it is better to find an alternative method to achieve the same. Recently, ultrathin supports have been utilized to develop high performance polymer composite membranes.^48–53^

**Scheme 1:**
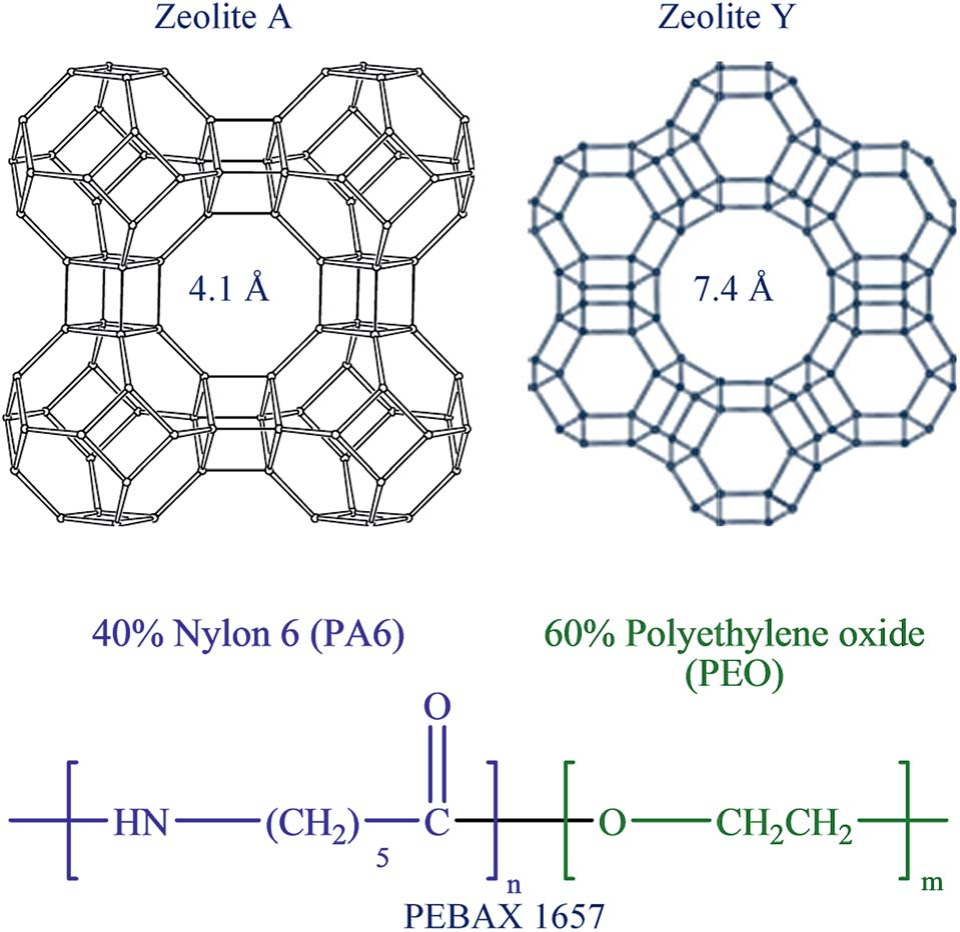
Zeolite framework structures representing the channel diameter of zeolites Y and A, whereas the chemical structure of PEBAX 1657 displays the composition of monomers.

In this reported paper, attempts have been made to improve the permeability as well as CO_2_ selectivity of the PEBAX-1657 membrane by improving the polymeric structure and controlling the free volume of the membrane. This work reflects that the free volume of the PEBAX-1657 membrane can easily be controlled by controlling the polymer chain conformation. Furthermore, to ascertain the structural as well as chemical reforms in the as-fabricated pristine PEBAX membrane elaborate diffraction, electron scanning, thermal, spectroscopic, *etc.*, analyses were conducted. Moreover, a number of experiments were performed to compare the selectivity of pristine PEBAX-1657 membranes with that of nano-molecular sieves (Scheme 1) embedded mixed matrix membranes (MMMs).

## 2. Experimental

### 2.1 Materials

Commercially available PEBAX® 1657 was used for membrane synthesis. The chemicals used for nano-size NaA and NaY zeolite molecular sieve synthesis, membrane preparation, and performance evaluation were as follows: tetramethylammonium silicate (TMAS) ((CH_3_)_4_N(OH)·2SiO_2_, 15–20 wt%, ≥99.99%, Aldrich), colloidal silica suspension (CSS) (SiO_2_, LUDOX®HS-30 colloidal silica, 30 wt% suspension in H_2_O, Aldrich), aluminium isopropoxide (Al(OCH(CH_3_)_2_)_3_, ≥98%, Aldrich), tetrametylammonium hydroxide (TMAOH) pentahydrate ((CH_3_)_4_N(OH)·5H_2_O, 98%, Alfa Aesar), tetrametylammonium bromide (TMABr) ((CH_3_)_4_N(Br), 98%, Sigma Aldrich), sodium hydroxide (min. 97%, Junsei Chemical Co. Ltd., Japan), and ethanol (EtOH) (99.9% absolute, OCI company Ltd.). All chemicals used in this study were of analytical grade and ultrapure water (0.054 μS cm^−1^) obtained from a μPure system (ROMAX, Human Science, Republic of Korea) was used throughout the experiments. Ultra-high purity grade CO_2_, O_2_, N_2_, and CH_4_ gases were used for single gas permeation experiments.

### 2.2 Zeolite molecular sieve synthesis

Zeolite molecular sieves (zeolite A (LTA type) and zeolite Y (FAU type)) having different porous characters (structure and size) and Si/Al ratios have been synthesized through liquid phase routes using a microwave hydrothermal reactor (CEM, Discover-909150, maximum power of 300 W). Detailed synthesis conditions with chemical compositions and their structural properties have been summarized in Table 1. After the synthesis, the resultant milky suspensions containing nano-size zeolite molecular sieves were cooled to room temperature and diluted with deionized water. The resulting nano-crystals were then separated from the mother liquor by high speed centrifugation at 20 000 rpm for 20 min at 4 °C followed by repeated re-dispersion in deionized water and centrifugation (washing step was repeated 5 times). To avoid the aggregation or coagulation of the nano-particles during drying, the finally collected wet zeolite particles were directly dispersed in DIW. The reported percentage of zeolite molecular sieves loaded on PEBAX-1657 membranes was directly obtained from the wet sample.

**Table 1:** Detailed reaction conditions for the as-synthesized zeolite nano-molecular sieves and their structural properties

Zeolite	Molar composition of the reaction mixture	Si/Al ratio	Silica source	Synthesis conditions				Structural properties	
				Aging		Crystallization		Pore size, Å	Ring size, membered
				Temp. °C	Time, h	Temp. °C	Time, h		
NaA	2.0SiO_2_ : 1.0Al_2_O_3_ : 2.2TMAOH : 0.16Na_2_O : 130.0H_2_O	1.0	TMAS	25	24	150	2	4.1	8
NaY	4.5SiO_2_ : 1.0Al_2_O_3_ : 5.0TMAOH : 2.5TMABr : 0.05Na_2_O : 250H_2_O	2.25	CSS	25	72	100	96	7.4	12

### 2.3 Membrane synthesis

Fig. S1, ESI† presents the stepwise preparation procedure for the pristine PEBAX and double layer PEBAX/nano-molecular sieve MMMs. PEBAX® 1657 pellets were dissolved in EtOH : H_2_O (70 : 30 wt%) solvent at 80 °C for 2 h with high speed stirring in a microwave hydrothermal reactor to obtain 2.5% PEBAX solution. Different thickness membrane films were obtained by pouring different amounts of polymer solution into a Teflon Petri dish (*ϕ* 85 mm). Subsequently, the polymer solution containing Teflon Petri dish was covered with filter papers (F1113 grade, Chmlab) to slow down the evaporation of solvent allowing the formation of a polymeric membrane with uniform thickness and better crystallinity. The polymeric solution was dried for 2 days at 40 °C. Once dried, the film was placed under vacuum for 24 h for complete solvent removal from the film. After this period, the film was allowed to cool to room temperature under vacuum. A 50 mm diameter circular sample was cut from the film and used for permeation tests, whereas, the PEBAX/nano-molecular sieve MMMs were fabricated by just casting the inorganic filler suspension in 1% PEBAX solution (EtOH : H_2_O: 70 : 30) on a dried PEBAX film as shown in Fig. S1, ESI† followed by drying. Casting of inorganic molecular sieves on the polymeric film instead of mixing with polymer solution was performed to avoid the void formation between the high concentration polymer and inorganic particles at their interface. The resultant membranes were denoted as P_75_, P_40_, P_22_, P_14_, P_63A_, and P_56Y_, where digits in the subscript represent the thickness of the membrane and letters molecular sieves.

### 2.4 Zeolite particles and membrane characterization

Powder X-ray diffraction studies of the nano-molecular sieves and PEBAX membranes were performed on a PANalytical: X'Pert PRO diffractometer with Cu-Kα radiation (*λ* = 1.5418 Å) and the data were collected in the 2*θ* range of 5–60° with a step size of 0.02° s^−1^. Phase identification for zeolites A and Y was performed with the help of JCPDS files for inorganic compounds (LTA #97-002-4901 and FAU # 98-003-4277). The morphological characteristics, particle size, and membrane thickness were evaluated with a scanning electron microscope (SEM, JEOL-JSM-7000F). The membrane samples were prepared by fracturing the membrane in liquid nitrogen and subsequent sputter coating of palladium. The average particle size and particle size distributions of the inorganic fillers were measured by light scattering analysis (Nanotrac Wave, Microtrac, Inc.) at 298 K. Transmission electron microscopy (TEM) images were obtained using an FEI Tecnai™ G^2^ F30 electron microscope, operating at 300 kV. The particle size distribution (PSD) in volume percent was calculated by using Microtrac FLEX 11 operating software. The thermal properties and glass transition temperatures (*T*_g_) of pristine PEBAX membranes were determined using a differential scanning calorimeter (DSC) (TGA/DSC 1 STAR^e^ system METTLER TOLEDO instruments). The measurements were carried out using a standard heating–cooling–heating procedure at a rate of 10 K min^−1^ in a nitrogen atmosphere. Furthermore, the chemical structure of the as-fabricated PEBAX membranes was characterized by using Raman spectra, which were recorded by using a LabRAM HR Evolution 800, Raman Spectrometer from Horiba Scientific (785 nm diode laser excitation). Fourier transform infrared spectroscopy (FTIR, ALPHA-Platinum ATR, Bruker) spectra of pristine PEBAX membrane samples were recorded in the range of 600–3600 cm^−1^ with a resolution of 1.4 cm^−1^ (64 scans) to assert the changes in the polymeric chain conformation. Water vapor adsorption/desorption experiments were performed at 298 K using an automatic adsorption measurement apparatus, BELSORP-max (BEL Japan, Inc.), and each sample was outgassed for 6 h at 423 K before analysis. A ∼66 point water vapor adsorption/desorption isotherm has been recorded and used for surface area and total pore volume calculation. The experimental values of membrane density were determined at 25 °C using Archimedes' principle with an electronic balance (Mettler Toledo XS205) equipped with a density measurement kit. An auxiliary liquid, silicon oil (*ρ*_o_ = 0.913 g cm^−3^), with known densities was used to evaluate the density, and to affirm the variation in fraction free volume (FFV). A piece (approximately 4 cm^2^) of each polymeric membrane was introduced into a sample pan and basket for weight measurement in air and the liquid phase, respectively. Considering some degree of hydrophilicity of the PEBAX membrane, two different fluid (de-ionized water and silicon oil) were selected to minimize the source of error due to the liquid–polymer interaction over the time scale of equilibration during weight measurement. The experimental values of density (*ρ*_p_) were calculated by using the following equation:

1 *ρ*_p_ = (*W*_air_/(*W*_air_ − *W*_liquid_))*ρ*_o_

where *W*_air_ and *W*_liquid_ are the membrane weight in air and in the liquid phase, respectively.

The fraction free volume (FFV) and specific free volume (SFV) of pristine PEBAX membranes were calculated using the following equations based on the density data:^24^

2 FFV = 1 − 1.3*ν*_w_*ρ*_p_

3 SFV = 1/*ρ*_p_ − 1.3*ν*_w_

where *ρ*_p_ is the density of PEBAX, and *ν*_w_ is the van der Waal's volume (0.590 cm^3^ g^−1^) of the repeat unit of PEBAX.^24^

### 2.5 Gas permeation measurement

**Fig. 1:**
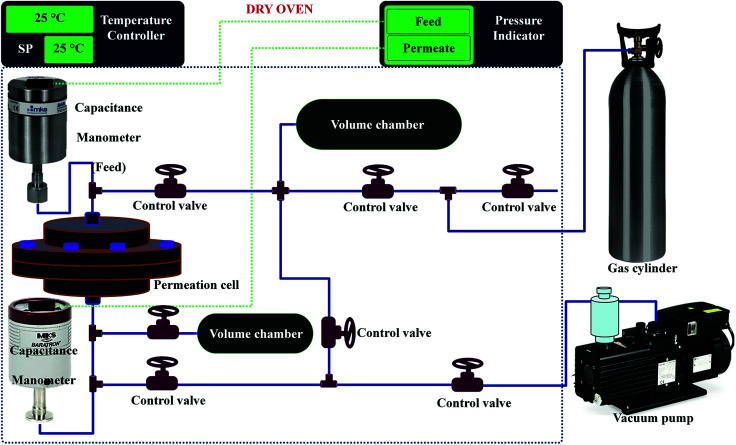
Schematic diagram of the experimental set-up (time-lag machine) for the single gas permeation.

The permeability of the polymeric membranes and MMMs were measured at room temperature (25 °C) and at a feed pressure of 2.7 bar using a single gas with a fixed-volume pressure increase instrument.^54^ The permeability was measured directly and the time lag method was applied to the recorded data to determine the diffusivity coefficient. The solubility coefficient was taken as the ratio of the permeability to the diffusivity coefficient. The schematic diagram of the permeation system is shown in Fig. 1. A flat sheet circular cell affixed with a mesh to support the membrane of effective area 15.2 cm^2^ was placed in a thermostat oven to control the temperature. Before analysis, the membranes were evacuated for at least 24 h to remove the previously adsorbed species. Moreover, to achieve accurate temperature apart from the gas cylinder, all other equipment/pipelines were installed inside a thermostat oven as shown in Fig. 1.

Gas transport in dense polymer membranes can be described by the solution–diffusion mechanism, in which the gas penetrants are absorbed into the membrane from the upstream feed and then diffuse through the membrane to the downstream permeate.^28,36^ Permeability (*P*) is an intrinsic property of the membrane material, which can be written as the product of diffusivity (*D*) and solubility coefficient (*S*):

4 *P*_i_ = *D*_i_*S*_i_

where *P*_i_, *D*_i_, and *S*_i_ represent the permeability (Barrer), diffusivity (cm^2^ s^−1^), and solubility (cm^3^(STP) cm^−2^ cmHg^−1^) coefficients of the penetrant component i, respectively.

In the ideal case of mass transport, *P*, *D*, and *S* are independent of feed/permeate pressure. Therefore, the selectivity of a polymer membrane for gas A over gas B will be the ratio of the gas permeability coefficients:

5 *α*_A/B_ = *P*_A_/*P*_B_

On using eqn (4) and (5) becomes;

6 *α*_A/B_ = (*D*_A_/*D*_B_)(*S*_A_/*S*_B_)

where *D*_A_/*D*_B_ and *S*_A_/*S*_B_ are the diffusivity and solubility selectivity, respectively.

## 3. Results and discussion

### 3.1 Molecular sieve characterization

**Fig. 2:**
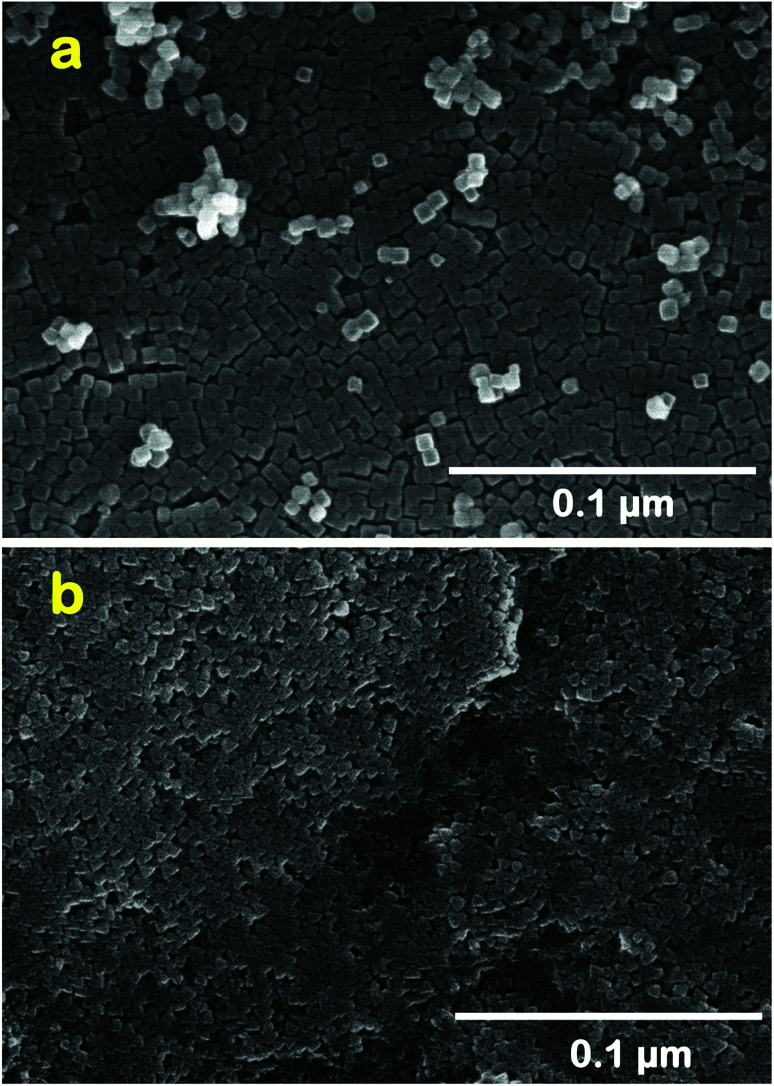
SEM micrographs of nano-sized zeolites A (a), and Y (b).

Nano-molecular sieve zeolites A and Y of different Si/Al ratios, shapes, pore sizes and channel structures (Scheme 1) have been successfully synthesized using a microwave hydrothermal reactor. SEM micrographs (Fig. 2a, b, S2, and S3, ESI†) of zeolite A and zeolite Y type molecular sieves show the formation of well-developed nanocrystals with a regular morphology. The zeolite A sample contains cube shaped nanocrystals of size around 55 nm, whereas the as-synthesized zeolite Y powder sample contains ∼40 nm octahedral crystals. The light scattering analysis (LSA) demonstrates the formation of uniform size crystals with narrow PSD (Fig. S4, ESI†). Moreover, Fig. S4 (ESI†) reveals that the average size of zeolite A cubic crystals is 60 nm, whereas that of zeolite Y octahedrons is 45 nm. Although the light scattering analyses just provide primary information about the nature and size of particles, the PSD curves presented in Fig. S4 (ESI†) for zeolite molecular sieves are in good agreement with microscopic analyses reported in Fig. 2a, b, S2, and S3, ESI.† Furthermore, the XRD patterns of zeolite A as well as zeolite Y (Fig. S5, ESI†) corresponded well with the earlier reported studies and JCPDS data file (LTA #97-002-4901 and FAU # 98-003-4277).^55,56^ The water vapor adsorption on both of the zeolite molecular sieves (Fig. S6, ESI†) displays a characteristic Type I shape in the IUPAC classification. These adsorption isotherms also highlight their greater water adsorption affinity as these microporous zeolite molecular sieves capture water vapor at a very low *P*/*P*_o_ value with steep uptake behavior. The experimentally calculated values of textural parameters of the zeolite molecular sieves reported in Table S1 (ESI†) reveal that zeolite Y has a higher surface area (1093.1 m^2^ g^−1^) as well as total pore volume (0.35 cm^3^ g^−1^) than zeolite A.

### 3.2 Membrane characterization

#### 3.2.1 Morphology evaluation

**Fig. 3:**
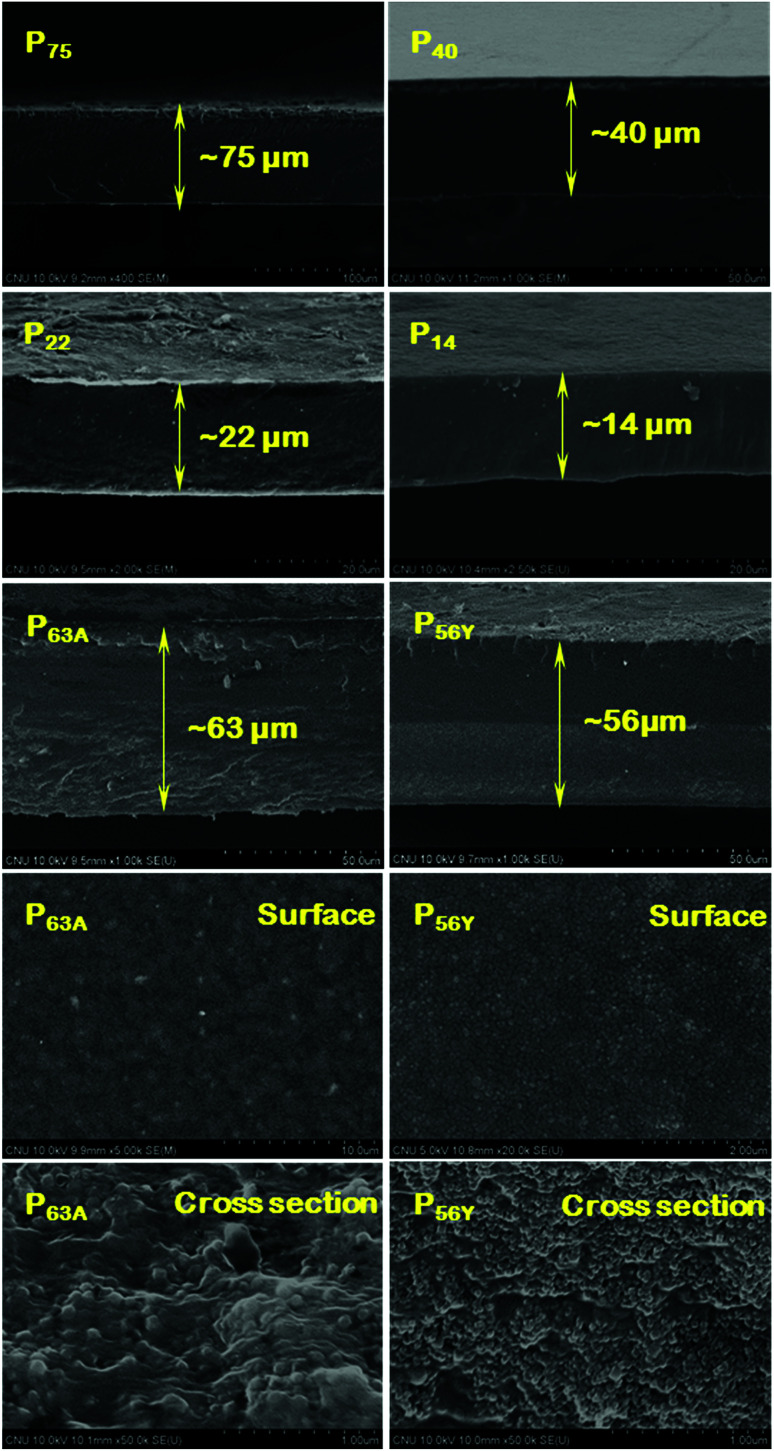
Cross sectional SEM micrographs of various thickness PEBAX-1657 membranes. Magnified view of the surface and cross section of P_63A_ and P_56Y_ membranes revealing the presence of zeolite A and Y nano-particles in PEBAX/nano-molecular sieve MMMs.

The SEM analysis of pristine PEBAX and PEBAX/nano-molecular sieve MMMs has been performed to investigate the surface smoothness, distribution of zeolite particles in the PEBAX polymer matrix, interfacial adhesion of the polymer and inorganic filler, accurate measurement of membrane thickness, *etc.* Photographic camera images and surface SEM micrographs of the membranes are presented in Fig. S7 (ESI†), whereas cross-sectional SEM micrographs and magnified surface and cross-sectional views of the MMMs are reported in Fig. 3. Photographic and surface SEM images display the clean and uniform growth of the pristine PEBAX membranes as well as MMMs. In the case of MMMs, the homogeneous distribution of nano-zeolite crystals of zeolites A and Y on the P_63A_ and P_56Y_ membrane surfaces can easily be observed. Moreover, these images also demonstrate the absence of nano-crystal agglomeration and interfacial void formation in the polymeric matrix. Furthermore, the cross-sectional SEM image of the membranes (Fig. 3) indicates that the uniform growth throughout the membrane thickness remains almost the same. The obtained membrane thickness value from the cross-sectional SEM images was used in the calculation of the gas permeation properties for each membrane. In addition, the cross-sectional images of P_63A_ and P_56Y_ membranes (Fig. 3) establish the successful growth of MMMs on the pristine PEBAX polymeric film. A little variation in the thickness of P_63A_ (63 μm) and P_56Y_ (56 μm) MMMs may arise from the difference in inorganic filler crystal sizes as zeolites A and Y crystals were 55 and 40 nm in size, respectively. Two distinct layers of almost the same thickness can be seen in these images (Fig. 3).

The magnified view of the surface and cross section of PEBAX/nano-molecular sieve MMMs containing 5 wt% polymer (Fig. 3) evidences that nano-zeolite crystals were comprehensively distributed in the PEBAX matrix without any aggregation and interfacial zeolite–polymer voids. The polymer was adhered well to the nano-zeolite crystals due to the rubbery nature of the continuous polymer phase and the presence of soft polymeric segments and smooth surfaced real nano-sized (around 50 nm) inorganic filler particles. The real nano-sized inorganic filler leads to the formation of a homogeneous and stable suspension in the polymeric solution as well as in the solvent which results in defect free MMMs.

#### 3.2.2 Crystallinity assessment

**Fig. 4:**
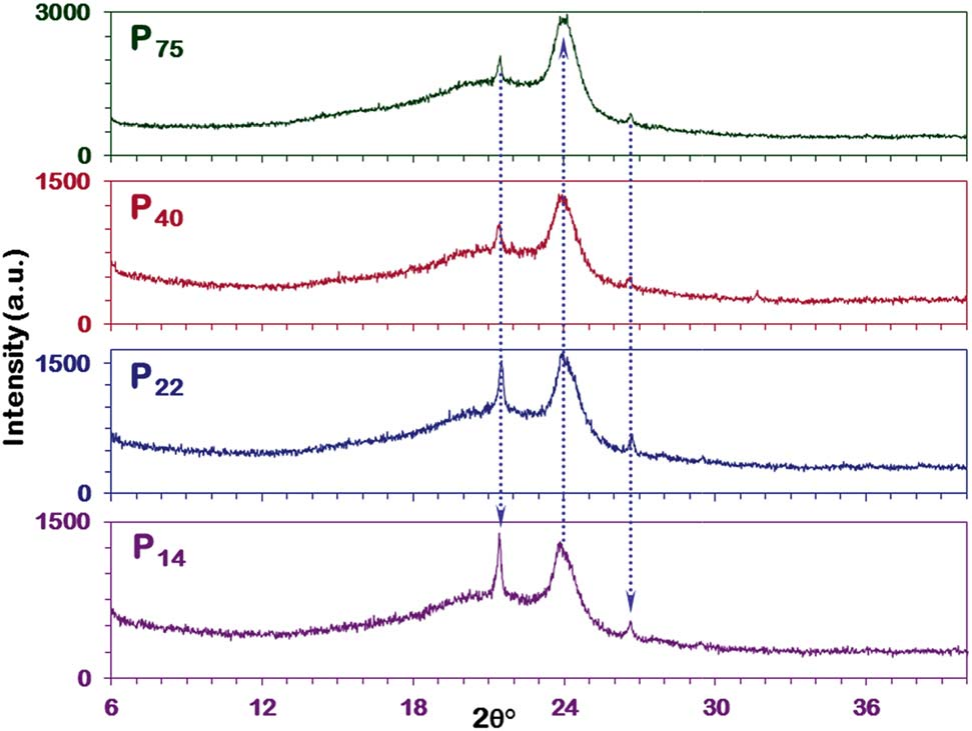
XRD patterns of various thickness PEBAX-1657 membranes. Direction of doted arrows shows the increase in respective peak intensities.

X-ray diffraction measurements were performed to evaluate the chain packing in the polymeric films. Representative XRD patterns for the pristine PEBAX membranes and MMMs of different thicknesses are collectively reported in Fig. 4 to compare the variation in diffraction patterns or the crystalline state with the polymerization or packing of PEO–PA units. PEBAX is a block copolymer with flexible polyether segments (polyethylene oxide) and a rigid block of polyamide (nylon 6); therefore XRD patterns reflect the semi-crystalline nature of various thickness polymeric films (Fig. 4 P_75_, P_40_, P_22_, and P_14_).^57^ The strong peak (21.4°) and a broad peak (at 24.0°) attributed to the crystalline PA6 phase correspond to *d*-spacing values of 4.15 and 3.70 Å, respectively. The decrease in the thickness of the polymeric film results in highly crystalline PA6 and PEO phases. Therefore, the sharp peak at a 2*θ* value of 26.7° (*d*-spacing value 3.34 Å) is attributed to the PEO phase. In addition to the characteristic PEO phase peak (26.7°), a low intensity peak for the same has also been observed at 29.5° in the XRD patterns of P_14_ and P_22_ pristine PEBAX samples. Moreover, the direction of arrows in Fig. 4 demonstrates the relative increase in peak intensities. On comparing the diffraction peaks corresponding to the PA6 phase we find that the peak intensity ratio of 21.4°/24.0° peaks increases with a decrease in the thickness of the polymeric membrane. Furthermore, the peak intensity of the PEO phase also increased with a decrease in membrane thickness. Hence, these diffraction patterns affirm that low thickness PEBAX membranes have better polymeric chain arrangement and crystallinity in comparison to thick membranes. It has been reported that nylon 6 (PA6) has two different crystalline structures, *i.e.*, α, and γ-forms. The molecules in the α crystal exhibit a fully extended, planar zigzag chain confirmation which is a thermodynamically stable structure and is well formed under slow crystallization while those in the γ-form crystal form a helical, metastable structure favored by rapid crystallization.^58,59^ Thus, these shifts in the diffraction peak intensity (Fig. 4) may be attributed to the chain packing in the copolymer films. Moreover, it is possible that the drying duration (2 days at 40 °C) is not sufficient for P_40_ and P_75_ membrane samples to dry completely under closed or controlled evaporation conditions. Therefore, understanding and controlling the crystal structures of polymers are pursued to gain insights into their mechanical properties and chain alignment.

To confirm the above findings, a comparison of XRD results with literature reported studies has been performed. On comparing Fig. 4 and S8, ESI,† we found that the as-fabricated membranes have distinct peaks for PA6 and PEO crystalline phases whereas in earlier reported studies no such distinction has been made. Most of the diffraction patterns in Fig. S8, ESI† show a broad peak ranging from 15–25° with a shoulder or small intensity peak, which implies the irregular packing of the polymer chains. The XRD patterns also evidence the formation of a metastable, helical γ-form type (PA6) structure in these PEBAX membranes. Moreover, these diffraction patterns (Fig. S8, ESI†) display a remarkably lower degree of crystallinity in comparison with P_14_, P_22_, P_40_, and P_75_ membranes (Fig. 4).

#### 3.2.3 Thermal analysis

According to the TGA results (Fig. S9a, ESI†), the thermal weight loss profiles for different thickness pristine PEBAX membranes follow the same trend and the pyrolysis of the membranes occurs between 350 and 450 °C which is in accordance with earlier reports.^32,60,61^ This deterioration of polymeric membranes is mainly associated with the decomposition reaction of the polymer precursors.

**Fig. 5:**
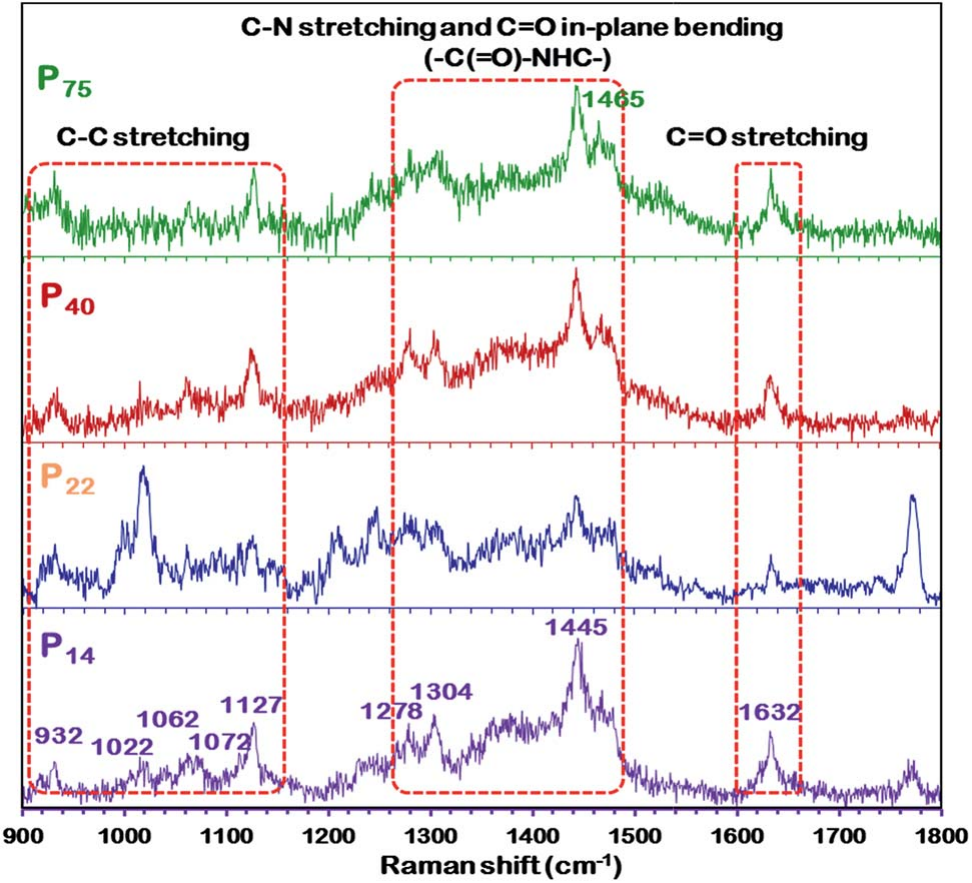
Raman spectra of different thickness PEBAX membranes.

Furthermore, to evaluate the thermal properties, polymeric chain rigidity, crystallization tendency, *T*_g_ and *T*_m_ (melting temperature) of polymers/segments, DSC analyses were performed. As reported in Fig. S9b (ESI†), the typical peaks of PEO and PA6 crystalline phases establish the semi-crystalline and phase separation characteristics of PEBAX membranes.^38^ In the case of the PEO segment, a small variation in the melting peak can be noticed. The area of the *T*_m_ PEO peak decreases with an increase in the membrane thickness whereas an increase in melting temperature has been observed which depicts the change in the crystallinity of the PEO segment. Moreover, a decrease in *T*_g_ values from −52.3 °C to −54.0 with a decrease in membrane thickness indicates the polymeric chain mobility and much regular arrangement. Furthermore, the *T*_g_ values reported in Fig. 5b for the PEO segment are much lower than previously reported values for pristine PEBAX membranes, where Xin *et al.*,^35^ Li *et al.*,^38^ Wang *et al.*^30^ and Rahman *et al.*^1^ reported −50.4 °C, −51.6 °C, −51.4 °C, and −51.0 °C for pristine PEBAX 1657 membranes, respectively. In addition to variation in the DSC profile of the PEO segment with a change in membrane thickness, a split in the *T*_m_ peak of the PA segment for the P_75_ membrane has also been observed which confirms the dual phase formation (α and γ PA6) during P_75_ membrane crystallization. Therefore, DSC observation affirms the XRD analysis findings.

#### 3.2.4 Chemical structure analysis

Vibrational spectroscopy, which is sensitive to the molecular confirmation of polymers, also demonstrated similar findings to those of XRD.^59,62^ As shown in Fig. 5, the spectrum of the high thickness P_75_ membrane exhibits different peak positions and intensities of characteristic peaks than that of the thin P_14_ membrane, which implies a change in the polymeric chain configuration. Additionally, there are obvious differences in the C–C stretching region (900–1150 cm^−1^) and the C–N–H bending region (1300–1350 and 1440–1490 cm^−1^), and in the intensity of the amide regions (Fig. 5). The amide band at 1632 cm^−1^ is primarily attributed to the CO stretch, while another amide mode is a combination of the C–N–H stretching and the C=O in-plane bending of the amide group (–C(O)–NHC–). The C–C stretching region is composed of four primary peaks, 1022, 1062, 1072 and 1127 cm^−1^. The 1062 and 1127 cm^−1^ peaks are indicative of an all-trans C–C backbone conformation while the 1080 cm^−1^ peak is attributed to the presence of gauche bonds in the polymeric film. The C–N–H bending region of the Raman spectrum is also sensitive to the conformation (planar or nonplanar) of the amide group. Bands observed in the regions of 1270–1350 and 1440–1490 cm^−1^ are indicative of different amide conformations. Furthermore, the disappearance or decrease in the intensity of the 1465 cm^−1^ peak in the spectrum of the P_14_ membrane evidences the change in the conformation of the polymer. If we go by the explanation given by Giller *et al.*^59^ and Stephens *et al.*^63^ for PA6, then we can also expect more than one energetically favorable crystalline structure (and/or chain conformations) in PEBAX membranes.

**Fig. 6:**
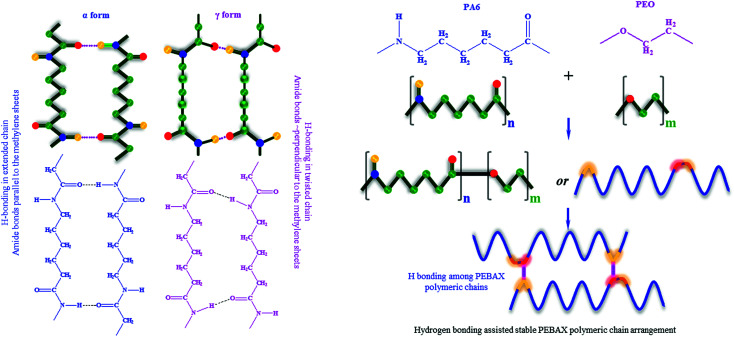
Two different classes of crystal structures formulated with different polymeric chain packing schemes in nylon 6. Schematic representation of PEBAX polymerization, and hydrogen bonding among polymeric chains leads to a stable polymeric configuration, high crystallinity and enhanced FFV.

Furthermore, nylon 6 is an important constituent of the PEBAX polymer and it imparts mechanical strength to PEBAX membranes. The –NH(CH_2_)_5_(CO)– repeating groups lead to a structure in which the peptide units (NH–CO) provide hydrogen bonding between polymer chains. Although nylon is crystalline, the presence of the crystalline lamella in an amorphous matrix makes it difficult to obtain precise crystallography.^64^ It has been well documented that nylon 6 exists in mainly two forms of regular crystal structures: α form (with amide bonds parallel to the methylene sheets) and γ form (with amide bonds perpendicular to the methylene sheets), as shown in Fig. 6.^58,59,64–66^ Out of these two forms, the α form is considered to be more stable than the γ form. If we go by these reported findings for nylon 6, then we can expect that the as-fabricated membranes have a different polymer conformation than that in earlier published reports. Thus, the polymeric chains in the crystalline zone of PEBAX may favor bonding among chains, and the nature of these folds affects the optimum chain spacing, chain structure/configuration, and connection between them. In simple words, the conformation of the PEBAX membrane has a high influence on its single gas permeation performance.

**Fig. 7:**
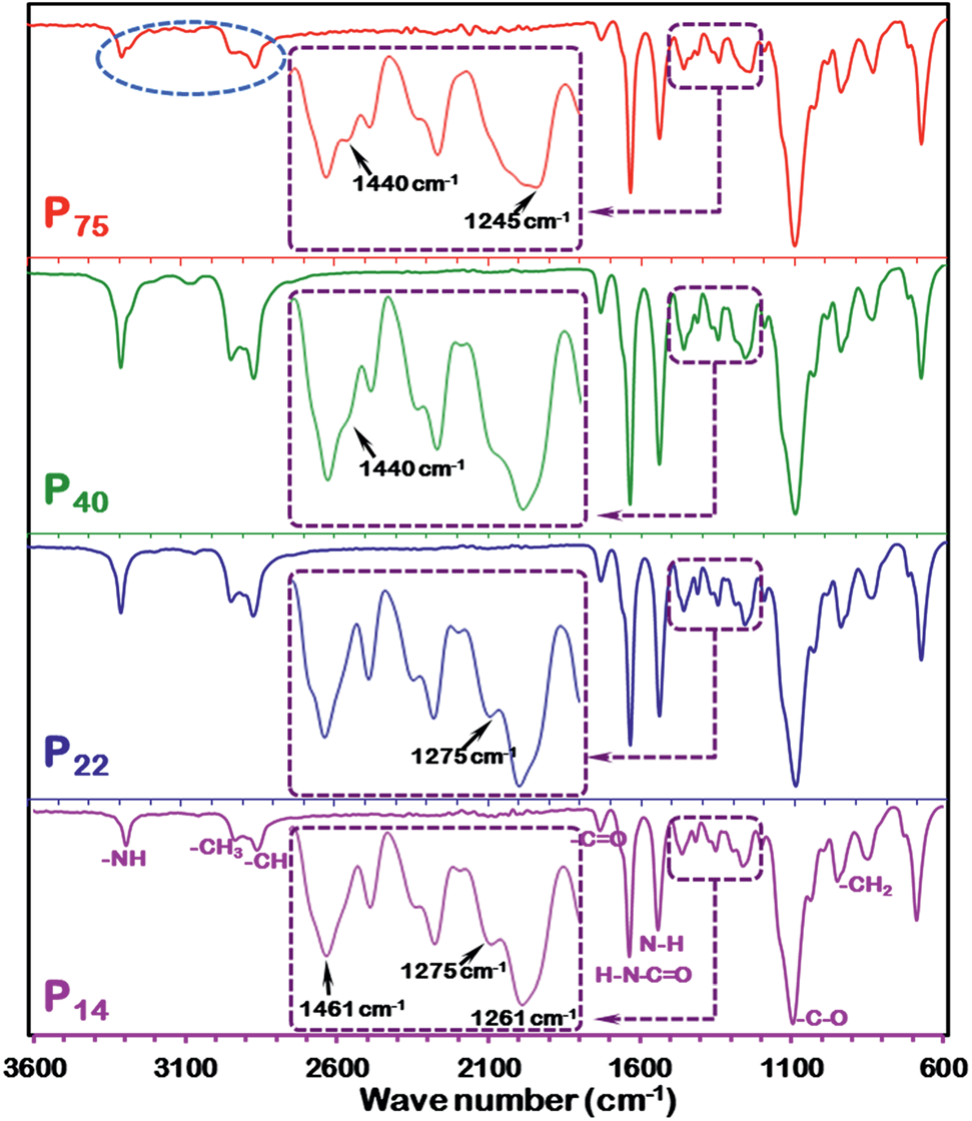
FTIR spectra of P_14_, P_22_, P_40_ and P_75_ PEBAX membranes. Inset spectra represent the variation in vibrational peaks in the range of 1200–1500 cm^−1^.

FTIR spectroscopy was used to explore the polymer conformation and interfacial interaction between the polymeric chains in the as-fabricated high permeability PEBAX membranes. Fig. 7 shows the FTIR spectra of the different thickness PEBAX membranes. The inset image in each spectrum reveals that there is a considerable difference between the vibrational peaks of low thickness and high thickness membranes (Fig. 7). A close look at the inset image in Fig. 7 P_14_ reveals a strong peak at 1461 cm^−1^ but with the increase in the thickness of the polymeric membrane in the P_44_ membrane sample, a shoulder appears which further changes to a low intensity peak (1440 cm^−1^) in the P_75_ sample. On the other hand, the 1275 cm^−1^ peak in P_14_ and P_22_ polymer films disappeared in P_40_ and P_75_ membranes which indicates a change in the polymeric chain arrangement with an increase in the thickness of the membrane. In addition to the disappearance of the 1275 cm^−1^ peak in the P_75_ membrane spectrum, a shift in the peak position (to 1245 cm^−1^), peak broadening and appearance of weak shoulder peaks have also been noticed. Moreover, vibrational peak patterns, especially in the 1200–1500 cm^−1^ wave number region of the as-fabricated PEBAX membranes, are unquestionably different from the spectra reported by Murali *et al.*,^26^ Wang *et al.*,^30^ Xiang *et al.*^32^ and Li *et al.*^38^ The appearance and disappearance of vibrational peaks in the 1200–1500 cm^−1^ wave number region demonstrate transformation in the polymer chain conformation and interfacial interactions as highlighted in Fig. 7.^58,67^ The interaction between the two different classes of crystal structures formulated with different polymeric chain packing schemes in nylon 6 can also be considered as a crucial factor for a stable polymeric chain configuration in PEBAX membranes. The FTIR spectrum of the P_75_ membrane also shows some change in the intensities and position shift of the symmetric stretching vibration peak of the –CH_3_ group highlighted by a dotted circle. Furthermore, the spectra reported in Fig. 7 reveal an unequivocally better polymeric chain arrangement and chemical structure than earlier reported spectra for pristine PEBAX 1657 membranes.^26,32^ The sharp and strong bands at 1732 and 1636 cm^−1^ represent C=O stretching vibration in the two types of amide groups, whereas 1541 cm^−1^ peaks correspond to N–H deformation of the PA6 segment.^38^ For the pristine PEBAX membrane, the characteristic peak at 1096 cm^−1^ is mainly attributed to the C–O stretching vibration of the PEO segment. As reported by Zheng and Xu,^67^ Ruiz-Hitzky and Aranda,^68,69^ and Papke *et al.*,^70^ the peaks at 949 and 848 cm^−1^ assigned to the CH_2_ rocking vibration of methylene groups in a gauche conformation indicate the possible helical structure of the PEO segment in the PEBAX membrane. From the Raman and FTIR spectra discussion, we can conclude that the polymer chain conformation, hydrogen bonding and interaction between polymeric chains play decisive roles in the free space and polymeric membrane performance.

#### 3.2.5 Free volume assessment

Free volume is useful for explaining the different aspects of polymers such as polymeric chain arrangements, mobility, and permeability but at the same time, it is relatively difficult to estimate with full accuracy. In the PEBAX membrane the PEO component mainly selectively interacts with polar gases such as CO_2_ to have high solubility selectivity towards polar gases. Ideally, based on the solution–diffusion model for dense membranes, the solubility selectivity due to the PEO segment should enhance the CO_2_ permeability if the diffusivity component PA6 is arranged well and not adversely affected by the PEO segment.^18^ As XRD and DSC studies affirmed the regular arrangement of the polymeric chain and high crystallinity PEBAX membrane formation, it becomes essential to evaluate the FFV and SFV. The FFV and SFV of pristine PEBAX membranes were estimated based on the group contribution method coupled with density measurements and the results are listed in Table 2. As shown, the FFV values of PEBAX membranes increase with a decrease in the membrane thickness which is favorable for enhancing the permeability.^38^ The P_14_ membrane of 14 μm thickness has the highest value of FFV as well as SFV. The obtained FFV values based on membrane density data are in good agreement with *T*_g_ values and crystalline character, as obtained from DSC and XRD results. In these terms, it seems clear that the glass transition temperature and the fraction of free volume have a distinct correlation. Virtually in the case of rubbery polymers, the FFV increases with decreasing *T*_g_.^71^ These observations indicate that the planar polymeric chain conformation with a stable polymeric structure formed under slow crystallization generates inter-chain spacing which results in significantly high FFV PEBAX membranes. The as-fabricated PEBAX membranes not only show a better polymeric structure but also have a high value of FFV in comparison to earlier reports.^16,38^

**Table 2:** Density, FFV and SFV values of different thickness pristine PEBAX membranes

Membrane	Density *ρ*_p_, g cm^−3^	Fractional free volume	Specific free volume, cm^3^
P_14_	1.129	0.134	0.119
P_22_	1.133	0.131	0.116
P_40_	1.144	0.123	0.107
P_75_	1.146	0.121	0.105

### 3.3 Single gas permeation performance assessment

#### 3.3.1 Gas permeability of pristine PEBAX membranes and MMMs

The single gas permeation performance of polymeric membranes and MMMs was measured by a single gas permeation test of CO_2_, N_2_, O_2_, and CH_4_. The polymeric membranes were different thickness PEBAX membranes whose thickness varies from 14–75 μm, and the MMMs were the polymeric membranes with 5 wt% zeolite nanoparticles (zeolites A and Y). The CO_2_, N_2_, O_2_, and CH_4_ permeability and their intrinsic selectivity for PEBAX and MMMs have been presented in Table 3. The tabulated values of permeability and permselectivity demonstrate a distinct relationship between CO_2_ permeability and the thickness of the membrane. It can be noticed that the high thickness PEBAX membrane (P_75_) displays a low value of permeability while the thin P_14_ membrane shows exceptionally high CO_2_ permeability. As can be seen in Table 3, the decrease of membrane thickness increases the permeability of CO_2_ while the permeability values of the other gases vary slightly. Consequentially, the ideal permeation selectivity values for CO_2_/N_2_, CO_2_/O_2_, and CO_2_/CH_4_ were increased with decreasing membrane thickness and stable PEBAX film formation. As there is a direct relationship between gas permeability and FFV, the increase of CO_2_ permeability and selectivity over the other gases can be attributed to increases in FFV. The increased selectivity can also be attributed to change in chain stiffness with membrane thickness variation. Unfortunately, the loading of zeolites A and Y onto PEBAX membranes shows a negative effect on membrane selectivity. In the case of MMMs (P_56Y_), an increase in gas permeation was observed for all the gases which results in low CO_2_ permselectivity. However, selectivity values are comparable with those of the pristine PEBAX P_75_ membrane.

**Table 3:** Pure gas permeation properties and permselectivity of the different thickness pristine PEBAX membranes

Membrane	Permeability (Barrer)				Selectivity			Gas flux (GPU)			
	CO_2_	N_2_	O_2_	CH_4_	CO_2_/N_2_	CO_2_/O_2_	CO_2_/CH_4_	CO_2_	N_2_	O_2_	CH_4_
P_14_	185	3.1	8.2	10	60	23	18	9.9	0.17	0.44	0.55
P_22_	148	2.7	7.1	8.8	56	21	17	5.7	0.11	0.28	0.35
P_40_	144	2.8	7.9	9.3	52	18	16	4.5	0.087	0.24	0.29
P_75_	88.5	2.3	6.5	7.9	39	14	11	1.2	0.030	0.087	0.11
P_63A_	54.3	1.5	—	5.3	35	—	10	0.72	0.020	—	0.070
P_56Y_	116	3.5	12	12	30	10	10	1.9	0.060	0.19	0.18

In order to obtain further insight into the role of the improved characteristics of the membrane in slow solvent evaporation and reduced thickness in gas permeation, the diffusivity and solubility data of the membranes were also measured using the time lag method. As shown in Table S2 (ESI†), the diffusion coefficient for CO_2_ gas increases for pristine PEBAX membranes as the thickness decreases. The diffusion coefficient of CO_2_ increases from nearly 1.2 × 10^−6^ cm^2^ s^−1^ for P_75_ to 2.6 × 10^−6^ cm^2^ s^−1^ for P_14_ pristine PEBAX membranes. The increase of FFV in both PEO and PA6 segments of pristine PEBAX membranes results in an enormous increase in CO_2_ diffusivity.^30^ Car *et al.*^72^ reported a CO_2_ diffusion coefficient of 4.6 × 10^−7^ cm^2^ s^−1^ at 30 °C and 600 mbar feed pressure. Kim *et al.*^73^ determined a CO_2_ diffusion coefficient of 1.52 × 10^−6^ cm^2^ s^−1^ by using a continuous flow technique at 25 °C and 3 atmosphere feed pressure. Furthermore, Wang *et al.*,^30^ Xin *et al.*^35^ and Reijerkerk *et al.*^36^ reported CO_2_ diffusion coefficients of 1.35 × 10^−6^ cm^2^ s^−1^, 1.43 × 10^−6^ cm^2^ s^−1^ and 7.9 × 10^−7^ cm^2^ s^−1^ for pristine PEBAX membranes, respectively. These comparative diffusivity results illustrated that the as-fabricated PEBAX membranes have a higher specific volume of the polymer in comparison to other reported pristine PEBAX membranes.

#### 3.3.2 Comparison with other pristine PEBAX membranes and PEBAX based MMMs

The CO_2_ gas permeation performance of the as-fabricated pristine PEBAX membranes was compared. The CO_2_ gas permeation performance of some representative pristine PEBAX, as well as PEBAX, based MMMs is compiled in Table S3 (ESI†) with detailed experimental conditions. Table S3 (ESI†) substantiates that the permeability as well as the selectivity of the as-fabricated pristine PEBAX membrane for CO_2_ and N_2_ separation is not only higher than that of literature reported pristine PEBAX membranes but also higher than that of MMMs. These results also show that we fabricated a high performance membrane without using any inorganic filler and with very low polymer concentration (2.5%). Furthermore, the thickness of the as-fabricated membrane (14 μm) is much lower than that of the other reported membranes. The enhanced selectivity of the as-fabricated pristine PEBAX membrane approves the high FFV and better polymerization of the polymeric film.^74^ The gas permeability for PEBAX based mixed matrix membranes reveals that addition of inorganic fillers to the polymeric film does not always boost the membrane performance. Furthermore, they also may have a negative effect on the adhesion of the polymer on the surface of the fillers which generates structural defects in the membrane. Furthermore, the addition of inorganic fillers some time induces polymeric chain destruction, active functional group blockage, *etc.*

## 4. Conclusions

Nanostructure zeolite molecular sieves (zeolites A and Y) were synthesized for MMM fabrication. Pristine PEBAX membranes of different thicknesses have been fabricated and their single gas permeation performance, polymeric conformation, and chemical structure have been evaluated extensively. The diffraction, thermal and spectroscopic studies assert that solvent evaporation kinetics plays a significant role in the observed polymeric conformation. Moreover, during evaporation, the polymeric film remains in contact with the vapor of the solvent which significantly affects the crystal structure of the resultant polymer. The single gas permeation results demonstrate the high potential of the reported polymeric structure optimization concept to significantly enhance the gas permeability. The optimal 14 μm thickness P_14_ membrane with high FFV shows very high CO_2_ permeability (184.7 Barrer) as well as CO_2_/N_2_ selectivity (59.7) at 2.7 bar and 25 °C. This paper also demonstrates that the permeability, as well as CO_2_ selectivity, can be improved by just reforming the polymeric structure without using any inorganic fillers and surfactants. It reveals that the permeability and selectivity improvement is mainly attributed to changes in the polymer conformation and total free volume. Thus, by improving the polymeric structure of PEBAX as well as by reforming the free-volume in the membrane, pristine PEBAX films can absorb CO_2_ molecules more favorably than N_2_ molecules. The pristine PEBAX membranes reported in this paper show even better performance than PEBAX based MMMs which encourages us to check the possibilities to fabricate high quality and greater CO_2_ separation performance membranes.

## Conflicts of interest

There are no conflicts to declare.

## Supplementary Material

NA-001-C9NA00170K-s001
