# Supplementary material for: A stable polymeric chain configuration producing high performance PEBAX-1657 membranes for CO2 separation
Source: Nanoscale Adv. 2019 May 14;1(7):2633–44. doi: 10.1039/c9na00170k (PMC9419191; doi:10.1039/c9na00170k)
Supplement: NA-001-C9NA00170K-s001 [file NA-001-C9NA00170K-s001.pdf]

Electronic Supplementary Material (ESI) for Nanoscale Advances.
This journal is © The Royal Society of Chemistry 2019

Electronic Supplementary Information (ESI)

Stable polymeric chain configuration producing high performance PEBAX-1657 membrane for CO_2 separation

Pankaj Sharma,* Young-Jin Kim, Min-Zy Kim, Syed Fakhar Alam, and Churl Hee Cho*

Graduate School of Energy Science and Technology, Chungnam National University, 99 Daehak-ro, Yuseong-gu, Daejeon 34134, Republic of Korea

*Corresponding author. Tel.: +82 42 821 8606; fax: +82 42 821 8839.

E-mail address: sharmapankaj47@yahoo.com (P. Sharma), choch@cnu.ac.kr (C.H. Cho)

This supplementary data file includes:

1. Figures S1-S9

2. Tables S1-S3

3. References

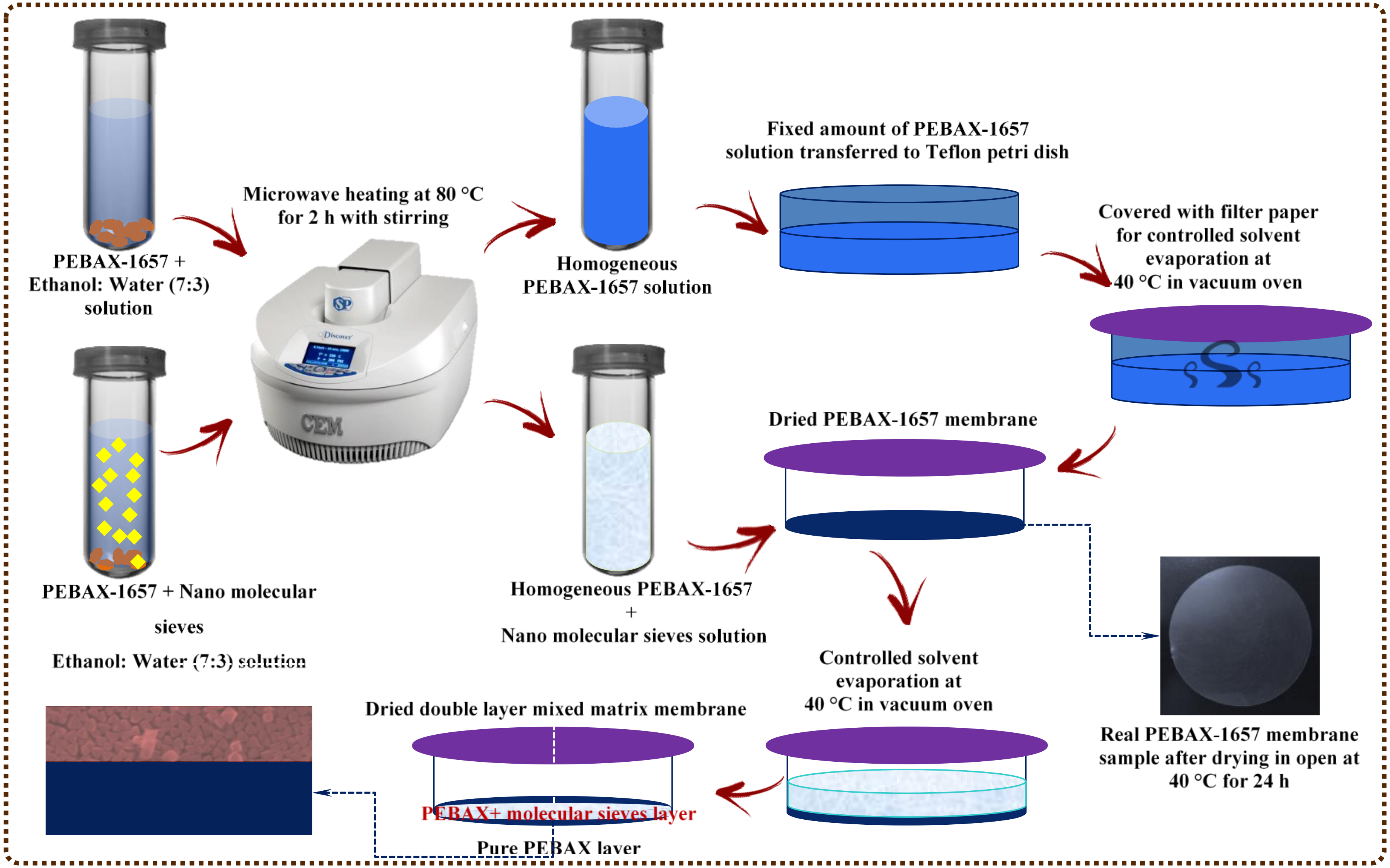

Fig. S1. Schematic description of pristine PEBAX and double layer PEBAX/nano molecular sieves MMMs preparation.

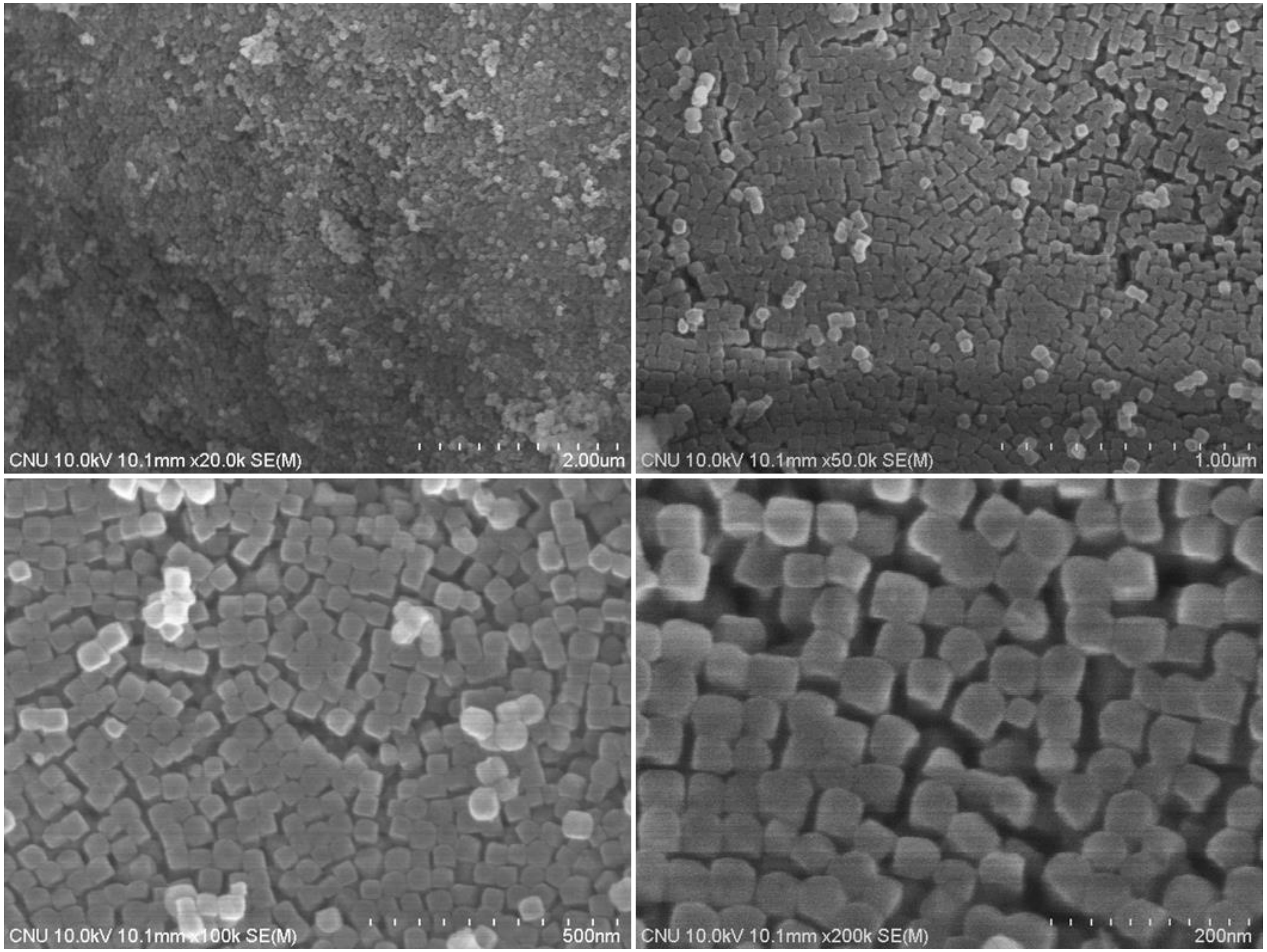

Fig. S2. Different magnification scanning electron micrographs of nano size zeolite A molecular sieve crystals.

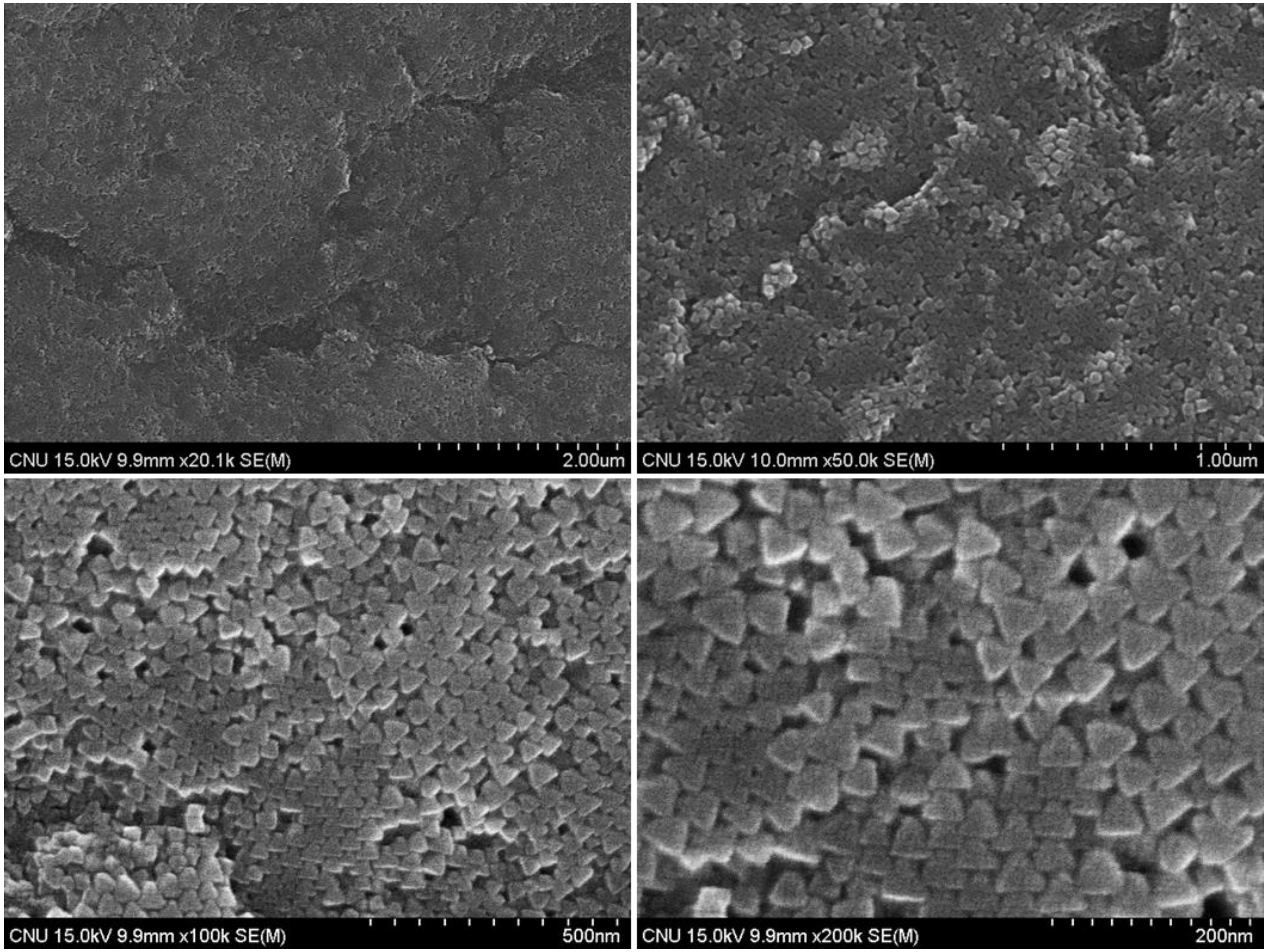

Fig. S3. Different magnification scanning electron micrographs of nano size zeolite Y molecular sieve crystals.

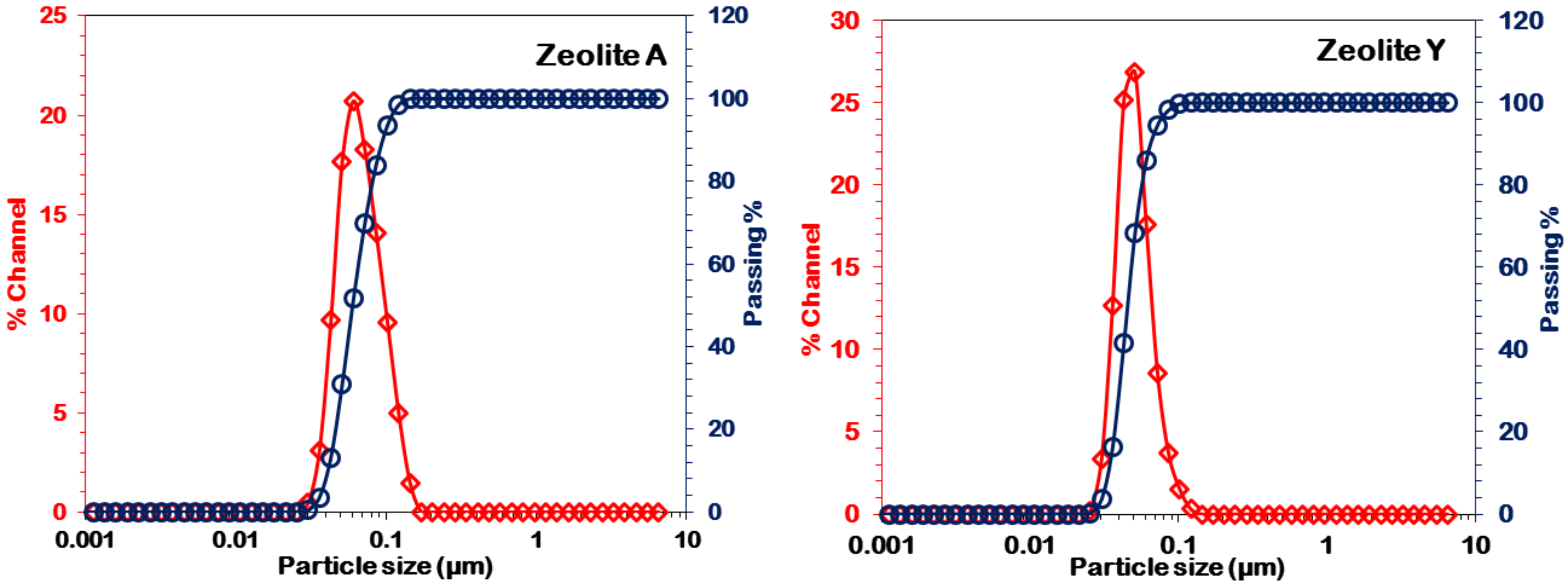

Fig. S4. PSD curves of as-synthesized zeolite molecular sieves A and Y.

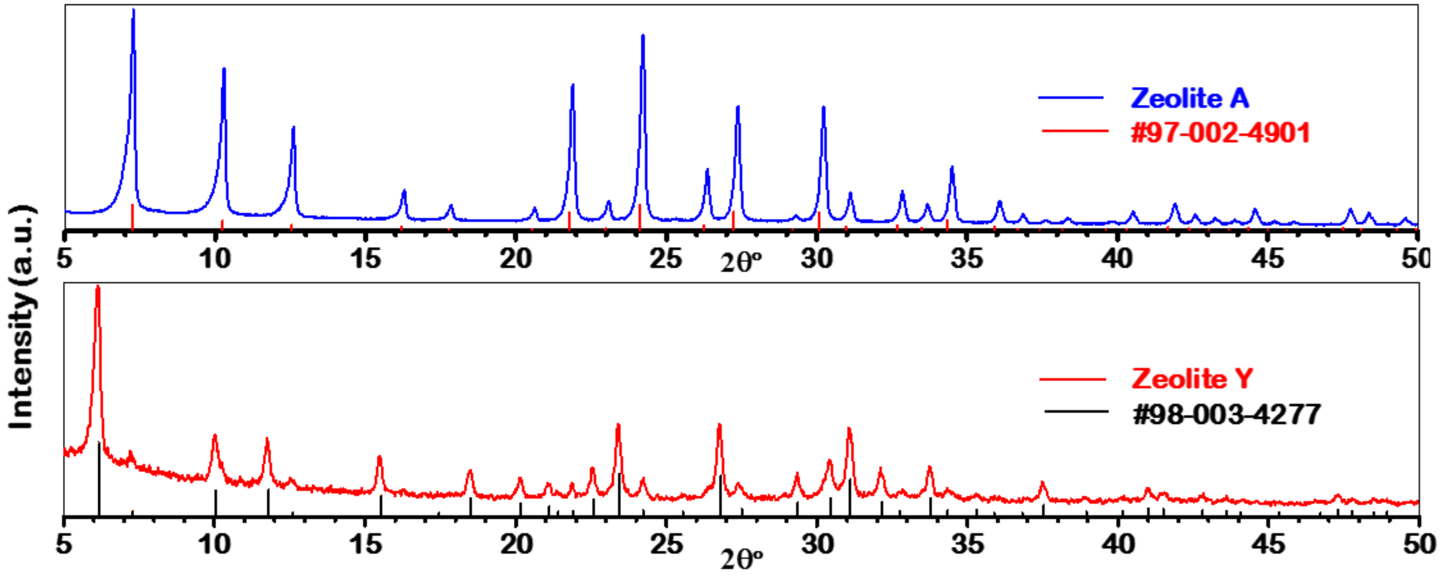

Fig. S5. X-ray diffraction patterns of as-synthesized zeolite molecular sieves A and Y along with their respective standard diffraction patterns.

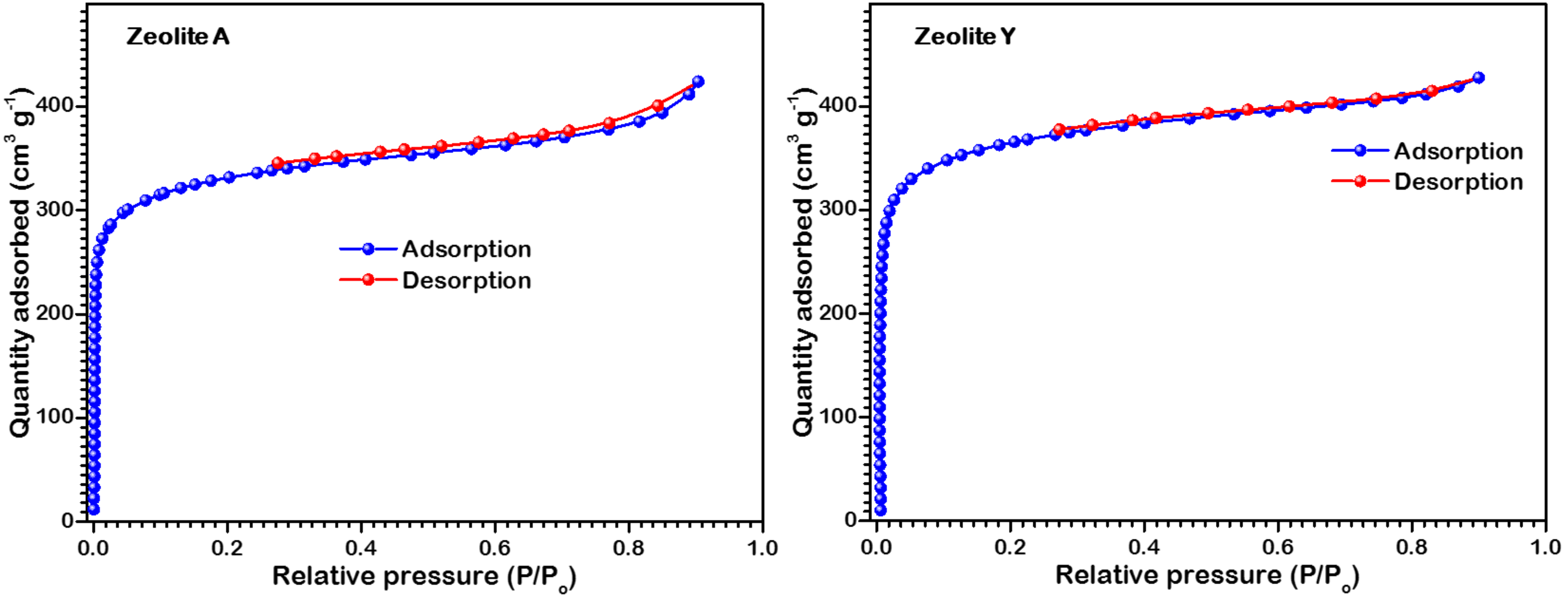

Fig. S6. Water vapor adsorption-desorption isotherms of nano size zeolites A and Y molecular sieve crystals at 298 K.

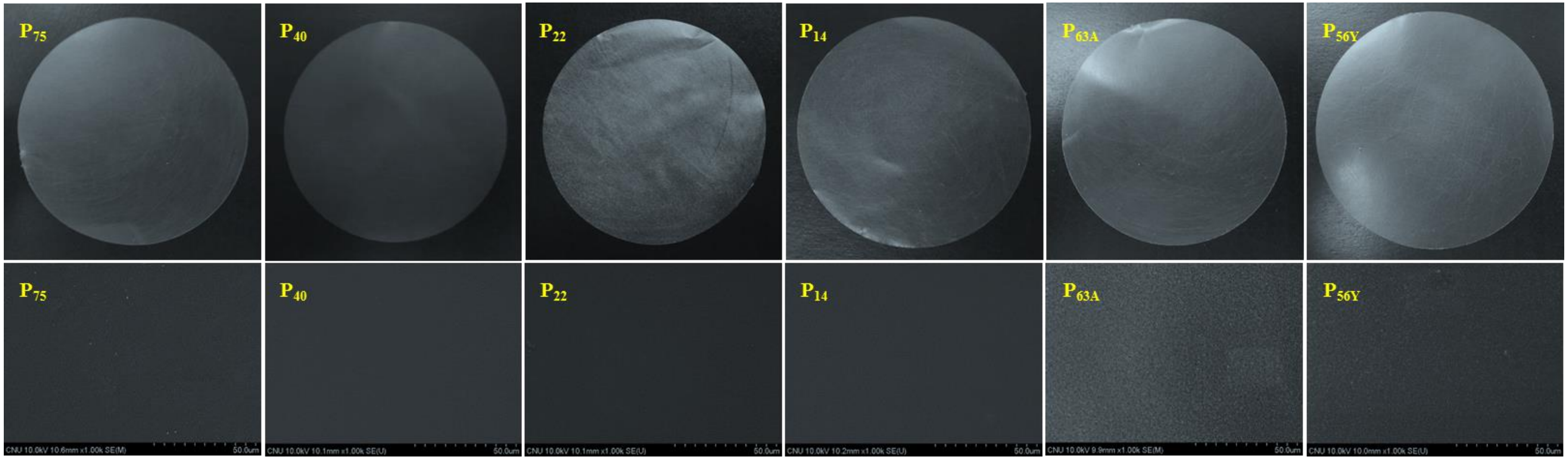

Fig. S7. Photographic camera images (top row), and surface SEM micrographs (bottom row) of various thickness PEBAX-1657 membranes.

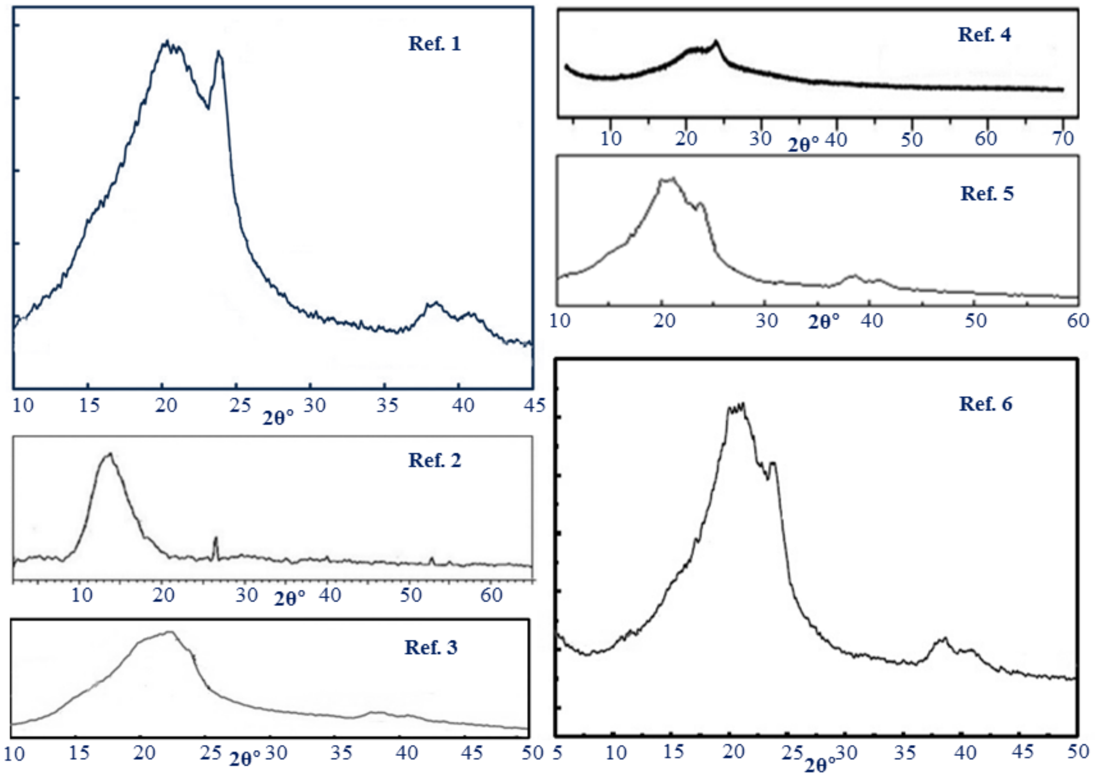

Fig. S8. Collective representation of X-ray diffraction patterns of pristine PEBAX-1657 membranes reported in literature to make a healthy comparison with diffraction patterns of the as-synthesized high crystallinity thin PEBAX membranes (**Fig. 4, main text**) (Adopted from ref.[1], and reproduced from refs.[2–6] with permission from the Elsevier).

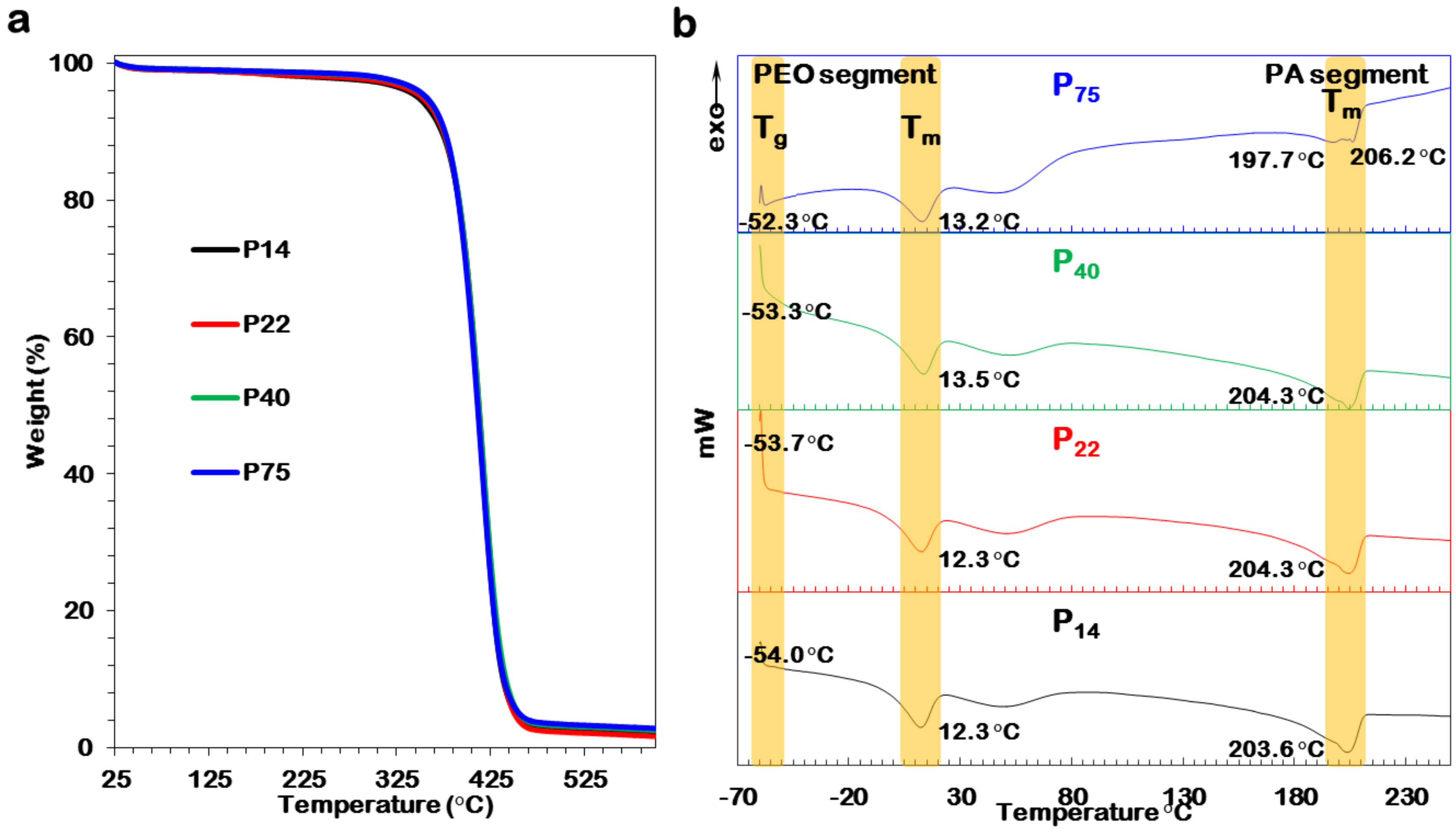

Fig. S9. (a) TGA curves, and (b) comparison of DSC profiles of various thickness PEBAX-1657 membranes.

Table S1. Textural parameters of zeolite molecular sieves determined by water vapor adsorption-desorption isotherms

Zeolite	Surface area, $m^2\ g^{-1}$	Pore volume (P/P_o= 0.90), $cm^3\ g^{-1}$
NaA	982.15	0.34
NaY	1093.10	0.35

Table S2. Gas diffusivity and solubility coefficients of the different thickness pure PEBAX membranes

Membrane	Diffusivity (cm^2 S^{-1})				Selectivity			Solubility x10^{-4} (cm^3(STP)/cm^2.cmHg)				Selectivity		
	CO_2	N_2	O_2	CH_4	CO_2/N_2	CO_2/O_2	CO_2/CH_4	CO_2	N_2	O_2	CH_4	CO_2/N_2	CO_2/O_2	CO_2/CH_4
P_{14}	2.60x10^{-6}	9.29x10^{-7}	2.25x10^{-7}	2.88x10^{-7}	2.80	11.56	9.03	65.48	3.02	35.73	33.16	21.63	1.83	1.97
P_{22}	1.47x10^{-6}	1.81x10^{-6}	1.89x10^{-5}	5.03x10^{-7}	8.12	0.78	29.22	9.73	1.46	0.41	18.85	6.65	23.85	0.52
P_{40}	1.35x10^{-6}	1.66x10^{-6}	1.01x10^{-6}	4.65x10^{-7}	0.81	1.34	2.90	100.76	1.68	7.92	21.57	59.95	12.72	4.67
P_{75}	1.21x10^{-6}	7.28x10^{-7}	7.63x10^{-7}	4.12x10^{-7}	1.66	1.59	2.94	70.37	3.31	8.62	20.19	21.26	8.16	3.48
P_{63A}	8.07x10^{-7}	2.67x10^{-7}	-	2.93x10^{-7}	3.02	-	2.75	67.31	5.77	-	17.99	11.67	-	3.74
P_{56Y}	1.24x10^{-6}	4.23x10^{-7}	1.26x10^{-6}	5.77x10^{-7}	2.93	0.98	2.15	93.64	8.32	9.13	19.36	11.25	10.25	4.84

Table S3. Comparison of literature reported pure PEBAX as well as MMM with as synthesize different thickness pure PEBAX membranes

Membrane	P_{CO_2}, Barrer	Selectivity			T, °C	Pressure, bar	Polymer Wt.%	Membrane thickness, μm	Ref.
		CO_2/N_2	CO_2/O_2	CO_2/CH_4					
PEBAX	80.0	49.0	-	-	30.0	1	3.0	-	[7]
PEBAX-40% PEG-GDMS-POSS-THF	160.0	40.4	-	-	30.0	1	3.0	-	[7]
PEBAX	80.0	70.0	-	-	21.0	2.3	7.5	50.0-90.0	[8]
PEBAX-30% PS colloid	44.0	66.0	-	-	21.0	2.3	7.5	50.0-90.0	[8]
PEBAX-5% SWNT	102.0	73.0	-	-	21.0	2.3	7.5	50.0-90.0	[8]
PEBAX	55.8	40.2	11.8	18.0	25.0	24.5	4.0	-	[2]
PEBAX-10% 4A zeolite	97.0	54.0	12.4	26.5	25.0	24.5	4.0	-	[2]
PEBAX	88.4	49.4	-	20.4	25.0	2	4.5	80.0-100.0	[3]
PEBAX-2% MWCNT	119.3	51.5	-	17.6	25.0	2	4.5	80.0-100.0	[3]
PEBAX	56.0	40.0	-	-	35.0	4	10.0	25.0	[4]
PEBAX-2% ATP	77.0	52.0	-	-	35.0	4	10.0	30.0	[4]
PEBAX	70.3	63.9	-	18.5	25.0	2	6.0	-	[9]
PEBAX-30% TEOS	41.8	64.3	-	16.1	25.0	2	6.0	-	[9]
PEBAX	115.0	48.0	-	18.0	30.0	2	3.0	65.0-85.0	[5]
PEBAX-Pro(Silica)	161.5	82.8	-	65.5	25.0	1	3.0	65.0-85.0	[5]
P_{14}	184.7	59.7	22.6	18.0	25.0	2.7	2.5	14.0	This work

References

1 Y. Li, S. Wang, H. Wu, J. Wang and Z. Jiang, *J. Mater. Chem.*, 2012, **22**, 19617–19620.

2 R. Surya Murali, A. F. Ismail, M. A. Rahman and S. Sridhar, *Sep. Purif. Technol.*, 2014, **129**, 1–8.

3 S. Wang, Y. Liu, S. Huang, H. Wu, Y. Li, Z. Tian and Z. Jiang, *J. Memb. Sci.*, 2014, **460**, 62–70.

4 L. Xiang, Y. Pan, G. Zeng, J. Jiang, J. Chen and C. Wang, *J. Memb. Sci.*, 2016, **500**, 66–75.

5 Q. Xin, Y. Zhang, T. Huo, H. Ye, X. Ding, L. Lin, Y. Zhang, H. Wu and Z. Jiang, *J. Memb. Sci.*, 2016, **508**, 84–93.

6 Y. Li, X. Li, H. Wu, Q. Xin, S. Wang, Y. Liu, Z. Tian, T. Zhou, Z. Jiang, H. Tian, X. Cao and B. Wang, *J. Memb. Sci.*, 2015, **493**, 460–469.

7 M. M. Rahman, S. Shishatskiy, C. Abetz, P. Georgopanos, S. Neumann, M. M. Khan, V. Filiz and V. Abetz, *J. Memb. Sci.*, 2014, **469**, 344–354.

8 B. Yu, H. Cong, Z. Li, J. Tang and X. S. Zhao, *J. Appl. Polym. Sci.*, 2013, **130**, 2867–2876.

9 A. Ghadimi, T. Mohammadi and N. Kasiri, *Ind. Eng. Chem. Res.*, 2014, **53**, 17476–17486.
